# Red and processed meat consumption and risk of bladder cancer: a dose–response meta-analysis of epidemiological studies

**DOI:** 10.1007/s00394-016-1356-0

**Published:** 2016-12-22

**Authors:** Alessio Crippa, Susanna C. Larsson, Andrea Discacciati, Alicja Wolk, Nicola Orsini

**Affiliations:** 1 Public Health Sciences, Karolinska Institutet, Tomtebodavagen 18A, 171 77 Stockholm, Sweden; 2 Unit of Biostatistics, Institute of Environmental Medicine, Karolinska Institutet, Nobels Vag 13, 171 77 Stockholm, Sweden; 3 Unit of Nutritional Epidemiology, Institute of Environmental Medicine, Karolinska Institutet, Nobels Vag 13, 171 77 Stockholm, Sweden

**Keywords:** Red meat, Processed meat, Bladder cancer, Dose–response, Meta-analysis

## Abstract

**Background/objectives:**

Several epidemiological studies have analyzed the associations between red and processed meat and bladder cancer risk but the shape and strength of the associations are still unclear. Therefore, we conducted a dose–response meta-analysis to quantify the potential association between red and processed meat and bladder cancer risk.

**Methods:**

Relevant studies were identified by searching the PubMed database through January 2016 and reviewing the reference lists of the retrieved articles. Results were combined using random-effects models.

**Results:**

Five cohort studies with 3262 cases and 1,038,787 participants and 8 cases–control studies with 7009 cases and 27,240 participants met the inclusion criteria. Red meat was linearly associated with bladder cancer risk in case–control studies, with a pooled RR of 1.51 (95% confidence interval (CI) 1.13, 2.02) for every 100 g increase per day, while no association was observed among cohort studies (*P* heterogeneity across study design = 0.02). Based on both case–control and cohort studies, the pooled relative risk (RR) for every 50 g increase of processed meat per day was 1.20 (95% CI 1.06, 1.37) (*P* heterogeneity across study design = 0.22).

**Conclusions:**

This meta-analysis suggests that processed meat may be positively associated with bladder cancer risk. A positive association between red meat and risk of bladder cancer was observed only in case–control studies, while no association was observe in prospective studies.

**Electronic supplementary material:**

The online version of this article (doi:10.1007/s00394-016-1356-0) contains supplementary material, which is available to authorized users.

## Introduction

Bladder cancer is the fifth most common cancer among men and the fourteenth among women with an estimated number of 429,000 cases worldwide in 2012 [1]. Bladder cancer is rather common in developed countries (North America and Europe), and it is more frequent among persons aged 75 or older [2]. Mortality rates have been stable over the last decade with 165,000 estimated deaths in 2012 [1]. A few risk factors have recently been linked to the etiology of bladder cancer. Apart from age and gender, cigarette smoking and specific occupational exposures are considered the most important risk factors [3, 4]. Identification of additional modifiable risk factors such as diet may enhance primary prevention.

Recently two meta-analyses summarized the body of evidence concerning red and processed meat consumption and risk of bladder cancer [5, 6]. Results from the review by Wang et al. [5] indicated an increased risk of bladder cancer of 17 and 10% for high red meat and high processed meat consumption, respectively. The more recent review by Li et al. [6], on the other hand, found a significant association for processed meat, with a 22% increased risk of bladder cancer for high consumption but not for red meat consumption. Both meta-analyses, however, were based only on contrasting risk estimates for the highest vs. the lowest category of meat consumption, and this has some limitations when the exposure distribution vary substantially across studies. In the review by Li et al. [6], one of the included studies [7] conducted in Uruguay, for instance, used 0–150 g/day of red meat consumption (median of 85 g/day) as the lowest category. In another study conducted in the USA [8], >58.5 g/day was the highest category for red meat consumption.

Our aim is to describe variation in bladder cancer risk across the whole range of the exposure distribution. A dose–response analysis is more efficient and less sensitive to heterogeneity of the exposure across different study populations. Therefore, we conducted a dose–response meta-analysis to clarify and quantify the potential association between red and processed meat and bladder cancer risk.

## Materials and methods

### Literature search and selection

Eligible studies were identified by searching the PubMed database through July 2016, with the terms [“bladder” AND (“carcinoma” or “cancer” or “tumor” OR “neoplasms”)] AND (“meat” or “beef” or “pork” or “lamb”). In addition, the reference lists of the retrieved articles were examined to identify additional reports. The search was restricted to studies written in English and carried out in human. We performed this meta-analysis accordingly to the Meta-Analysis of Observational Studies in Epidemiology (MOOSE) guidelines [9]. Two authors (A.C. and A.D.) independently retrieved the data from studies on the association between red and processed meat and risk of bladder cancer. Discrepancies were discussed and resolved.

Studies were eligible if they met the following criteria: (1) the study was a cohort or case–control study; (2) the exposure of interest was red meat and/or processed meat; (3) the outcome was incidence of bladder cancer; (4) the authors reported measures of association (hazard ratio, relative risk, odds ratio) with the corresponding confidence intervals for three or more categories for red or processed meat consumption. In case of multiple reports on the same study population, we included only the most comprehensive or recent one.

### Data extraction

From each study, we extracted the following information: first author’s surname, year of publication, study design, country where the study was conducted, study period, exposure definition, unit of measurement, number of cases, study size, confounding variables, and measure of associations for all the categories of meat consumption together with their confidence intervals. Given the low prevalence of bladder cancer, the odds ratios were assumed approximately the same as the relative risks (RRs). When several risk estimates were available, we included those reflecting the greatest degree of adjustment.

### Statistical analysis

We used the method described by Greenland and Longnecker [10] and Orsini et al. [11] to reconstruct study-specific trend from aggregated data, taking into accounts the covariance among the log RR estimates. Risk estimates from studies not reporting information about the number of deaths and study size did not allow reconstruction of the covariance and were assumed independent. Potential nonlinear associations were assessed by use of restricted cubic splines with three knots located at the 10th, 50th, and 90th percentiles of the exposure distribution. An overall *P* value was calculated by testing that the regression coefficients were simultaneously equal to zero. A *P* value for nonlinearity was obtained by testing that the coefficient of the second spline term was equal to zero [12].

Since studies used different units to express meat consumption (e.g., servings/day, grams/day, grams per 1000 kcal/day), we converted frequency of consumption using 120 and 50 g as the average portion sizes for red and processed meat, respectively. We chose those values in accordance with previous meta-analyses on meat consumption and other types of cancer [13, 14] and results from both the Continuing Survey of Food Intakes by Individuals [15] and the European Prospective Investigation into Cancer and Nutrition [16]. Meat consumption reported in grams per 1000 kcal/day was converted to g/day using the average energy intake reported in the original articles. Within each exposure category, the median or mean value was assigned to the corresponding RRs. If not reported, we assigned the midpoint of the upper and lower boundaries as average consumption. If the upper bound of the highest category was not reported, we assumed that the category had the same width as the contiguous one. When RRs were reported only for single food items (e.g., separately for beef and pork), we combined them using a fixed-effects model and used the pool estimate as summary measure.

A random-effects meta-analysis was adopted to acknowledge heterogeneity across study findings. Statistical heterogeneity was further assessed by using the *Q* test (defined as a *P* value less than 0.10) and quantified by *R*_*b*_ statistic [17]. Meta-regression models were employed to explain residual heterogeneity. Differences in dose–response curves between subgroups of studies were tested as described by Berlin et al. [18]. Evaluation of goodness-of-fit for the final models was assessed using the set of tools presented by Discacciati et al. [19]. Publication bias was investigated using the Egger asymmetry test [20].

We performed sensitivity analyses (1) excluding studies where red meat definition included also some items of processed meat; (2) excluding studies that did not adjust for energy intake; (3) evaluating alternative average portion sizes for red meat (100 and 140 g) and processed meat (30 and 70 g) consumption. All statistical analyses were conducted with the dosresmeta [21] and metafor [22] packages in R (R Foundation for Statistical Computing, Vienna, Austria) [23]. *P* values less than 0.05 were considered statistically significant.

## Results

### Literature search

**Fig. 1:**
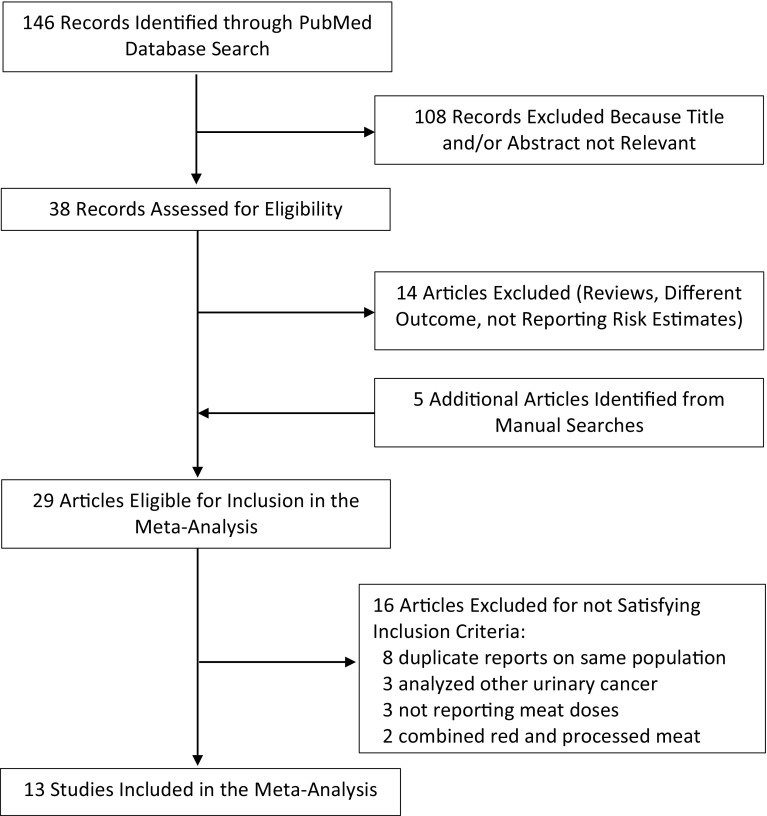
Selection of studies for inclusion in a meta-analysis of red and processed meat consumption and risk of bladder cancer 1966–2016

The search strategy identified 146 articles, 108 of which were excluded after review of the title or abstract (Fig. 1). Of the 38 eligible papers 14 were excluded because they did not meet the inclusion criteria (not original articles, outcome different from bladder cancer, or not reporting risk estimates with their confidence intervals). The reference lists of the remaining 24 articles were checked for additional pertinent reports, and 5 additional papers were identified. We further excluded 16 additional articles: 8 presented duplicated publications [24–31]; 3 analyzed bladder and other urinary cancer together [32–34]; 3 did not report enough data for a dose–response analysis [35–37]; and 2 did not report results for red or processed meat consumption [16, 38]. Thus, the meta-analysis included 13 independent epidemiological studies [7, 8, 31, 39–49].

### Study characteristics

The main characteristics of the 13 epidemiological studies included in the meta-analysis are presented in Table 1. Five cohort studies [39–43] with 3262 cases and 1038,787 participants and 8 cases–control studies, of which 4 population-based [8, 44, 46, 47] and 4 hospital-based [7, 45, 48, 49], with 7009 cases and 27,240 participants evaluated the relation between red and/or processed meat and risk of bladder cancer. Two articles [39, 49] reported results only for red meat, while one [44] only for processed meat. Definition of meat and red meat varied across studies but generally included beef, veal, pork, and lamb for red meat, and bacon, ham, salami, sausages, and hot dogs for processed meat. Two cohort studies [39, 40] included also processed meat in the definition of red meat, and one study [42] included only results for specific food items. One study [44] reported results only for liver intake and was not included in the analysis of red meat. Another study [45] analyzed preserved meat consumption and, given the limited range of exposure (up to 1/week), was excluded from the analysis of processed meat.

**Table 1:** Characteristics of epidemiological studies of meat consumption and risk of bladder cancer in a meta-analysis, 1966–2016

References	Study name	Country	Study period	No. of cases	Study size	Exposure definition	Exposure contrasts	RR (95% CI)	Adjustment variables
*Cohort*									
Jakszyn [39]	European Prospective Investigation into Cancer and Nutrition	Europe		1001	481,419	Red meat (fresh and processed)	Red meat		Age, gender, center, educational level, BMI (as continuous variable), smoking status, lifetime intensity of smoking (number of cigarettes per day), time since quitting or duration of smoking, and total energy intake
							57.86–91.42 g/day versus 0–57.86 g/day	1.2 (0.96–1.49)	
							91.42–130.63 g/day versus 0–57.86 g/day	1.14 (0.91–1.41)	
							130.63–754.79 g/day versus 0–57.86 g/day	1.15 (0.9–1.45)	
Ferrucci [40]	NIH-AARP Diet and Health Study	USA	1995–2004	854	300,933	Red meat (bacon, beef, cold cuts, ham, hamburger, hot dogs, liver, pork, sausage, and steak) and processed meat (bacon, sausage, luncheon meats, ham, and hot dogs)	Red meat		Age (continuous, years), sex, smoking (never, quit 10 years ago, quit 5–9 years ago, quit 1–4 years ago, quit <1 year ago, or 20 cigarettes/day, 20–40 cigarettes/day, >40 cigarettes/day), and intakes of fruit (continuous, cup equivalents/1000 kcal), vegetables continuous, cup equivalents/1000 kcal), beverages (continuous, mL/day; sum of beer, coffee, juice, liquor, milk, soda, tea and wine), and total energy (continuous, kcal/day)
							20.9 g per 1000 kcal versus 9.5 g per 1000 kcal	0.99 (0.78–1.25)	
							30.7 g per 1000 kcal versus 9.5 g per 1000 kcal	1.05 (0.83–1.33)	
							42.1 g per 1000 kcal versus 9.5 g per 1000 kcal	0.97 (0.77–1.23)	
							61.6 g per 1000 kcal versus 9.5 g per 1000 kcal	1.22 (0.96–1.54)	
							Processed meat		
							4.3 g per 1000 kcal versus 1.6 g per 1000 kcal	1.09 (0.85–1.39)	
							7.4 g per 1000 kcal versus 1.6 g per 1000 kcal	1.1 (0.86–1.41)	
							12.1 g per 1000 kcal versus 1.6 g per 1000 kcal	1.28 (1.01–1.62)	
							22.3 g per 1000 kcal versus 1.6 g per 1000 kcal	1.10 (0.86–1.40)	
Larrson [41]	Swedish Mammography Cohort and the Cohort of Swedish Men	Sweden	1998–2007	485	82,002	Red meat (meatballs or hamburger, beef, pork, veal, kidney, and liver) and processed meat (sausage, ham, salami, and cold cuts)	Red meat		Age, sex, education, smoking status, pack-years of smoking, and total energy intake
							1–4 servings/week versus 0–3 servings/month	1.11 (0.81–1.52)	
							≥5 servings/week versus 0–3 servings/month	1.00 (0.71–1.41)	
							Processed meat		
							1–4 servings/week versus 0–3 servings/month	0.87 (0.68–1.11)	
							≥5 servings/week versus 0–3 servings/month	1.91 (0.80–1.28)	
Michaud [42]	Health Professionals Follow-Up Study and the Nurses’ Health Study	USA	1986–2002 and 1976–2002	808	135,893	Red meat (hamburger, beef, pork, lamb as main or mixed dish) and processed meats (bacon, hot dogs, sausage, salami, bologna)	Hamburger		Age, caloric intake (quintiles), and pack-years of smoking and for geographic region
							0 serving/month versus 1–3 servings/month	0.99 (0.72–1.36)	
							1 serving/week versus 1–3 servings/month	0.86 (0.68–1.08)	
							2–4 servings/week versus 1–3 servings/month	0.91 (0.70–1.17	
							Beef, pork, or lamb (main dish)		
							0 serving/month versus 1–3 servings/month	1.35 (0.94–1.96)	
							1 serving/week versus 1–3 servings/month	1.01 (0.78–1.33)	
							2–4 servings/week versus 1–3 servings/month	1.11 (0.85–1.45)	
							≥5 servings/week versus 1–3 servings/month	0.93 (0.57–1.52)	
							Beef, pork, or lamb (sandwich or mixed dish)		
							0 serving/month versus 1–3 servings/month	1.06 (0.79–1.43)	
							1 serving/week versus 1–3 servings/month	0.83 (0.65–1.06)	
							2–4 servings/week versus 1–3 servings/month	1.26 (0.98–1.63)	
							≥5 servings/week versus 1–3 servings/month	0.95 (0.51–1.75)	
							Hamburger:		
							0 serving/month versus 1–3 servings/month	1.07 (048–2.41)	
							1 serving/week versus 1–3 servings/month	1.13 (0.80–1.60)	
							2–4 serving/week versus 1–3 servings/month	0.96 (0.66–1.38)	
							Beef, pork, or lamb (main dish):		
							0 serving/month versus 1–3 servings/month	2.28 (0.88–5.92)	
							1 serving/week versus 1–3 servings/month	1.35 (0.76–2.39)	
							2–4 servings/week versus 1–3 servings/month	1.23 (0.71–2.11)	
							≥5 servings/week versus 1–3 servings/month	1.01 (0.56–1.65)	
							Beef, pork, or lamb (sandwich or mixed dish)		
							0 serving/month versus 1–3 servings/month	1.61 (0.92–2.81)	
							1 serving/week versus 1–3 servings/month	1.03 (0.75–1.41)	
							2–4 servings/week versus 1–3 servings/month	0.92 (0.66–1.27)	
							≥5 servings/week versus 1–3 servings/month	0.83 (0.40–1.71)	
							Processed meats (e.g., sausage, salami, bologna)		
							1–3 servings/month versus 0 serving/month	0.98 (0.76–1.25)	
							1 serving/week versus 0 serving/month	0.94 (0.71–1.23)	
							2–4 servings/week versus 0 serving/month	0.98 (0.74–1.30)	
							≥5 servings/week versus 0 serving/month	1.09 (0.71–1.69)	
							Bacon		
							1–3 servings/month versus 0 serving/month	1.08 (0.86–1.37)	
							1 serving/week versus 0 serving/month	1.09 (0.84–1.41)	
							2–4 servings/week versus 0 serving/month	1.10 (0.82–1.49)	
							≥5 servings/week versus 0 serving/month	1.63 (1.02–2.62)	
							Hot dog		
							1–3 servings/month versus 0 serving/month	1.02 (0.83–1.25)	
							1 serving/week versus 0 serving/month	1.02 (0.78–1.34)	
							2–4 servings/week versus 0 serving/month	0.86 (0.58–1.27)	
							Processed meats (e.g., sausage, salami, bologna)		
							1–3 servings/month versus 0 serving/month	1.07 (0.76–1.52)	
							1 serving/week versus 0 serving/month	1.25 (0.86–1.84)	
							2–4 servings/week versus 0 serving/month	0.98 (0.65–1.46)	
							≥5 servings/week versus 0 serving/month	0.81 (0.40–1.63)	
							Bacon		
							1–3 servings/month versus 0 serving/month	0.90 (0.65–1.25)	
							1 serving/week versus 0 serving/month	1.06 (0.74–1.51)	
							2–4 servings/week versus 0 serving/month	1.00 (0.67–1.51)	
							≥5 servings/week versus 0 serving/month	1.48 (0.70–3.16)	
							Hot dog		
							1–3 servings/month versus 0 serving/month	0.91 (0.66–1.24)	
							1 serving/week versus 0 serving/month	0.89 (0.63–1.27)	
							2–4 servings/week versus 0 serving/month	0.77 (0.47–1.24)	
Nagano [43]	Life-Span Study	Japan	1979–1993	114	38,540	Red meat and processed meat (ham, sausage)	Red meat		Age, gender, radiation dose, smoking status, education level, body mass index, and calendar time
							2–4 servings/week versus 0–1 serving/week	0.68 (0.45–1.04)	
							5+ servings/week versus 0–1 serving/week	1.13 (0.53–2.19)	
							Ham and sausage		
							1 serving/week versus 0 serving/week	0.54 (0.31–0.92)	
							2+ servings/week versus 0 serving/week	0.73 (0.42–1.28)	
*Case–control*									
Catsburg [44]		USA	1987–1996	1660	3246	Processed meat (fried bacon, ham, salami, pastrami, corned beef, bologna, other lunch meats, hot dogs and polish sausage)	Processed meat		Age, sex, BMI (underweight/normal <25, overweight 25–30, obese >30), race/ethnicity (non-Hispanic white/Hispanic/black or other), education (high school/1- to 4-year college/grad school), history of diabetes (yes/no), total vegetable intake per day, vitamin A intake (IU per day), vitamin C intake (mg per week), carotenoid intake (mcg per day), total servings of food per day, smoking duration (years smoked) and smoking intensity (cigarettes per day)
							1–2 times a week versus < Once a week	0.96 (0.76–1.23)	
							3 times a week versus < Once a week	1.11 (0.87–1.41)	
							4–6 times a week versus < Once a week	1.23 (0.96–1.58)	
							>1 time a day versus < Once a week	0.97 (0.74–1.27)	
Isa [45]		China	2005–2008	487	956	Red meat and preserved meat	2–4 times/week versus ≤1 times/week	1.20 (0.90–2.10)	Sex, age (categorical), smoking status (categorical), smoking duration (continuous), smoking amount (continuous), and other food groups
							≥5 times/week versus ≤1 times/week	1.80 (1.10–3.00)	
							Preserved meat		
							<1 times/month versus never	1.60 (1.00–2.80)	
							1–3 times/month versus never	1.70 (0.90–3.10)	
							1 times/week versus never	2.20 (1.00, 4.7)	
Wu [46]		USA	2001–2004 and 2002–2004	1171	2535	Red meat (beef, veal, pork, and lamb) and processed meat (ham, bacon, sausage, hot dog, cold cuts, turkey sausages and hot dogs, and poultry cold cuts)	Red meat		Gender, age (0–54, 55–64, 65–74, 75+), region, race (White/other), Hispanic status, smoking status (never, occasional, former, current), usual BMI (continuous), and total energy (kcal per day—continuous)
							27.6 g per 1000 kcal versus 17.2 per 1000 kcal	0.97 (0.76–1.24)	
							37.4 g per 1000 kcal versus 17.2 per 1000 kcal	1.04 (0.81–1.33)	
							53 g per 1000 kcal versus 17.2 per 1000 kcal	1.14 (0.89–1.46)	
							Processed meat		
							6.1 g per 1000 kcal versus 2.9 per 1000 kcal	1.01 (0.78–1.30)	
							10.1 g per 1000 kcal versus 2.9 per 1000 kcal	1.19 (0.92–1.53)	
							18.4 g per 1000 kcal versus 2.9 per 1000 kcal	1.28 (1.00–1.65)	
Lin [8]		USA	1999	884	1762	Red meat (beef, veal, lamb, pork and game) and processed meat (hot dogs or franks, sausage or chorizo)	Red meat		Age, sex, ethnicity, smoking status, pack-year of smoking, energy intake, total vegetable intake, total fruit intake, and BMI
							0.55–1.10 once versus <0.55 once	1.17 (0.87–1.58)	
							1.11–2.05 once versus <0.55 once	1.47 (1.09–1.99)	
							≥2.06 once versus <0.55 once	1.95 (1.41–2.68)	
							Processed meat:		
							0.11–0.28 once versus <0.11 once	0.88 (0.66–1.18)	
							0.29–0.61 once versus <0.11 once	0.98 (0.73–1.31)	
							≥0.62 once versus <0.11 once	1.03 (0.76–1.39)	
Aune [7]		Uruguay	1996–2004	255	2287	Red meat (fresh meat including beef and lamb) and processed meat (hot dogs, sausages, ham, salami, saucisson, mortadella, bacon and salted meat)	10–40 g/day versus 0–10 g/day	1.01 (0.70–1.46)	Age, sex, residence, education, income, interviewer, smoking status, cigarettes per day, duration of smoking, age at starting, years since quitting, alcohol, dairy foods, grains, fatty foods (butter, eggs, custard, cake), fruits and vegetables, fish, poultry, mate drinking, BMI, and energy intake
							>40–258.8 versus 0–10 g/day	1.43 (0.93–2.20)	
Hu [47]		Canada	1994–1997	1209	6248	Red meat (beef, pork, lamb as a main or mixed dish and hamburger) and processed meat (hot dogs, smoked meat, corned beef, bacon and sausage)	Red meat		Age group (20–49, 50–59, 60–69, 70–76), province, education, body mass index (<25, 25–29.9, ≥30), sex, alcohol use (g/day), pack-year smoking, total of vegetable and fruit intake, and total energy intake
							2.1–3.94 times/week versus ≤2 times/week	1.20 (1.00–1.60)	
							3.95–5 times/week versus ≤2 times/week	1.20 (090–1.50)	
							≥5.42 times/week versus ≤2 times/week	1.30 (1.0–1.70)	
							Processed meat:		
							0.95–2.41 times/week versus ≤0.94 times/week	1.20 (1.10–1.60)	
							2.42–5.41 times/week versus ≤0.94 times/week	1.50 (1.10–1.90)	
							≥5.42 times/week versus ≤0.94 times/week	1.60 (1.20–2.10)	
Closas [48]		Spain	1998–2001	912	1785	Red meat (beef, veal, lamb, pork) and processed meat	Red meat:		Age (<55, 55–64, 65–69, 70–74, >74 years old), gender, region, smoking status (never, occasional, former, current), duration of smoking (<20, 20–<30, 30–<40, 40–<50, ≥ 50 years) and quintiles of fruit and vegetable intake
							(20–32) g per 1000 kcal versus <20 g per kcal	1.10 (0.80–1.50)	
							(33–43) g per 1000 kcal versus <20 g per kcal	1.10 (0.80–1.50)	
							(44–58) g per 1000 kcal versus <20 g per kcal	1.00 (0.70–1.30)	
							(>58) g per 1000 kcal versus <20 g per kcal	0.80 (0.60–1.10)	
							Processed meat:		
							(4–9) g per 1000 kcal versus <4 g per kcal	1.40 (1.00–1.90)	
							(10–12) g per 1000 kcal versus <4 g per kcal	1.20 (0.90–1.70)	
							(13–18) g per 1000 kcal versus <4 g per kcal	1.20 (0.80–1.60)	
							(>18) g per 1000 kcal versus <4 g per kcal	1.20 (0.90–1.70)	
Tavani [49]		Italy	1983–1996	431	8421	Red meat (beef, veal and pork)	Red meat		Age, year of recruitment, sex, education, smoking habits and alcohol, fat, fruit and vegetable intakes
							3–6 times/week versus ≤3/week	1.40 (1.20–1.80)	
							≥6 times/week versus ≤3 times/week	1.60 (1.20–2.10)	

Only 3 studies [40, 46, 48] considered different cooking methods and doneness levels for meat consumption. None of them found evidence of an association between preparation methods and bladder cancer. Different units were used to report meat consumption: servings/week (7 studies), grams per 1000 kcal per day (3 studies), and grams per day (3 studies). Five studies were conducted in the USA, 4 in Europe, and 1 each in Canada, Uruguay, China, and Japan. All the studies were carried out in both men and women, and only one study [42] reported results separately by gender. All the studies provided measure of associations adjusted for age, gender, and smoking. Four studies did not adjust for energy intake [43–45, 49]. Other common adjusting variables were other food groups (8 studies), BMI (6 studies), education (6 studies). Additional covariates were less consistent across studies.

### Association between red meat consumption and risk of bladder cancer

We found a statistically significant association between red meat consumption and risk of bladder cancer (*P* = 0.009; *P* nonlinearity = 0.74) (Online Resource 1). The summary RR for an increment of 100 g per day of red meat was 1.22 (95% CI 1.05, 1.41). There was substantial between-studies heterogeneity (*R*_*b*_ = 67%, *P* < 0.01). Egger’s regression test did not suggest the presence of substantial publication bias (*P* = 0.14).

**Fig. 2:**
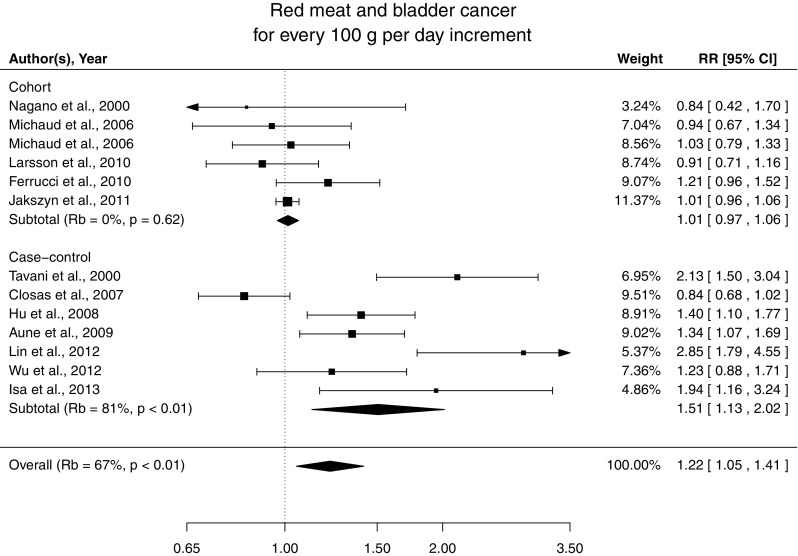
Relative risks of bladder cancer with 100 g per day increment in red meat consumption separately for cohort and case–control studies

There was statistical heterogeneity according to study design (*P* for heterogeneity = 0.02). The pooled RR restricted to the cohort studies was 1.01 (95% CI 0.97, 1.06) for an increment of 100 g per day of red meat with no significant heterogeneity (*R*_*b*_ = 0%, *P* = 0.62) (Figure 2). The deviance test did not detect lack of fit (*D* = 24, *df* = 18, *P* = 0.17). In case–control studies, the corresponding pooled RR was 1.51 (95% CI 1.13, 2.02) with substantial heterogeneity among studies (*R*_*b*_ = 81%, *P* < 0.01) and overall indication of poor fit (*D* = 44, *df* = 18, *P* < 0.01).

No differences were found according to study location (*P* for heterogeneity = 0.7), units of measurement (*P* for heterogeneity = 0.38), and selection of controls (*P* for heterogeneity = 0.65). Excluding those studies with also processed meat in the definition of red meat, the pooled RRs were 1.27 (95% CI 1.03, 1.57) overall and 0.95 (95% CI 0.82, 1.11) restricted to cohort studies. The pooled RR for an increment of 100 g of red meat per day was 1.14 (95% CI 0.99, 1.31) based on studies that adjusted for energy intake. In the sensitivity analysis for alternative average portion sizes of red meat, the results did not substantially change. The pooled RR for an increment of 100 g of red meat per day was 1.27 and 1.19 for assigned portions of 140 g per day and 100 g per day, respectively.

For an increment of four servings per week of red meat (120 g per servings), the summary RR of bladder cancer was 1.15 (95% CI 1.03, 1.27) overall, 1.01 (95% CI 0.98, 1.04) for cohort studies, and 1.32 (95% CI 1.08, 1.62) for case–control studies.

### Association between processed meat consumption and risk of bladder cancer

**Fig. 3:**
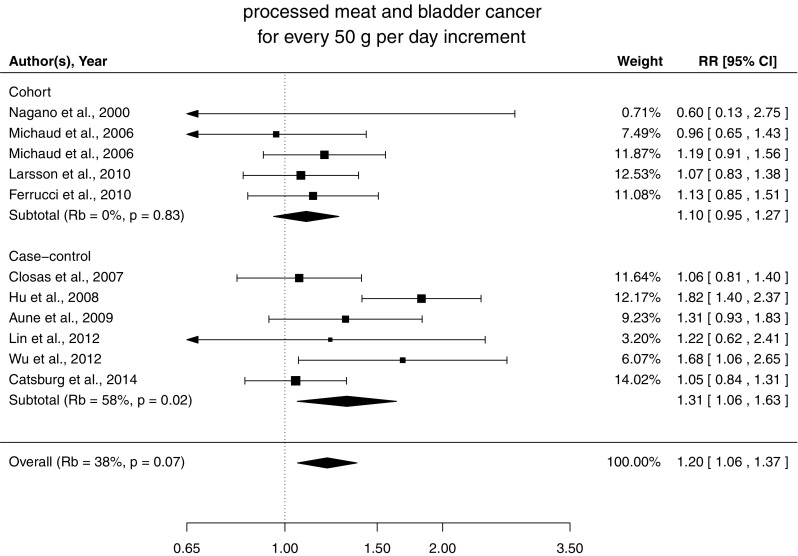
Relative risks of bladder cancer with 50 g per day increment in processed meat consumption separately for cohort and case–control studies

We found a statistically significant association between processed meat intake and bladder cancer with no departure from linearity (*P* = 0.005, *P* nonlinearity = 0.92) (Online Resource 2). Every 50 g increase in processed meat per week was associated with a 20% (95% CI 6, 37) increase in risk of bladder cancer with moderate heterogeneity (*R*_*b*_ = 38%, *P* = 0.07). Egger’s regression test did not detect publication bias (*P* = 0.21). No evidence of lack of fit was observed (*D* = 43, *df* = 34, *P* = 0.14). The test did not detect significant differences between case–control and cohort studies (*P* for heterogeneity = 0.22). Stratified analysis provided a RR of 1.10 (95% CI 0.95, 1.27) and 1.31 (95% CI 1.06, 1.63) for cohort and case–control studies, respectively (Fig. 3).

The associations were similar across strata of study location (*P* for heterogeneity = 0.68), units of measurement (*P* for heterogeneity = 0.71), and selection of controls (*P* for heterogeneity = 0.46). Exclusion of studies that did not adjust for energy intake provided a pooled RR of 1.24 (95% CI 1.07, 1.43). Similar results were observed for alternative average portion sizes of 30 g per day and 70 g per day with pooled RR, respectively, of 1.14 and 1.36 for an increment of 50 g per day of processed meat.

For an increment of four servings per week of processed meat (50 g per servings), the summary RR of bladder cancer was 1.11 (95% CI 1.03, 1.20) overall, 1.06 (95% CI 0.97, 1.15) for cohort studies, and 1.17 (95% CI 1.03, 1.32) for case–control studies.

## Discussion

Findings from this dose–response meta-analysis of five cohort and eight case–control studies suggest that processed meat consumption is positively associated with risk of bladder cancer. An increment of 50 g of processed meat per day was associated with 20% increased risk of bladder cancer. Red meat consumption was associated with bladder cancer only in case–control studies, with a 51% increased risk of an increment of 100 g per day, while no association was observed among the prospective studies.

Meat, in particular processed meat, is a potential risk factor for several cancers, with the most convincing evidence for colorectal cancer [50]. In 2015, the International Agency for Research on Cancer classified processed meats as carcinogenic to humans (Group 1) and red meat as probably carcinogenic to humans [51]. The contribution of meat to the etiology of bladder cancer may be explained by different mechanisms, given that many nutrients are excreted through the urinary tract [52]. The most established mechanism involves the formation of endogenous nitrosamines from nitrites that are particularly abundant in processed meats [53]. Experimental studies have shown that some nitrosamine metabolites induce bladder tumors in rodents [54, 55]. Further support for at potential role of nitrosamines in bladder carcinogenesis is that cigarette smoking is a strong risk factor for bladder cancer and tobacco smoke is a main source of exogenous exposure to nitrosamines. Consumption of red meat could potentially increase the risk of bladder cancer through heterocyclic amines and polycyclic aromatic hydrocarbons, which can be generated from high temperature cooking [56]. Heterocyclic amines and polycyclic aromatic hydrocarbons have been consistently shown to be carcinogenic in animal studies [56, 57].

A direct comparison with the results of previous reviews [5, 6] is difficult since they were based on study-specific risk estimates for high versus low categories of meat consumption, which varied substantially across studies. The directions of the associations, however, were consistent, even though an association was found only for processed meat in the meta-analysis by Lin et al. [6]. As in the review by Wang et al. [5], case–control studies provided stronger risk estimates as compared to prospective studies.

Among several potential explanations, case–control studies generally assess the exposure after diagnosis, and therefore, recall bias may lead to differential misclassification between cases and controls. Considering the limited knowledge of the role of meat consumption on the development of bladder cancer [44], such classification errors are likely to be similar among cases and controls. On the other hand, half of the control studies used hospital-based controls which may inflate the pooled association in case controls have been recruited for conditions linked with changes in meat consumption. Although based on limited number of studies, we did not observed differences in results between hospital-based and population-based case–control studies. Different participation rates related to exposure or severity of diseases may also be a selection bias among case–control studies. In addition, the time between diagnosis and the exposure assessment is generally shorter for case–control studies; hence, it may not reflect long-term exposure because of changes in dietary patterns. On the other hand, in cohort studies participants may alter their dietary intake during the follow-up, which may bias results toward the null hypothesis of no association. One of the included cohort studies [42] analyzed repeated dietary measurements over time and observed stronger associations when using cumulative update date and when removing participant who had change their meat consumption.

Strength of this review is the dose–response analysis, which better takes into account the quantitative nature and heterogeneity of the exposure. In our analysis, all the information about meat consumption, including intermediate categories, contributed to the pooled associations. Another strength is the large number of cases that provided high statistical power to detect associations of moderate magnitude. Lastly, no evidence of publication bias was observed.

This meta-analysis also had potential limitations. Pooling results from epidemiological studies do not solve the problem of residual confounding, which inherently affects individual studies. All of the included studies, however, adjusted for main known risk factors for bladder cancer such as age, gender, and smoking, and some studies also adjusted for energy intake, BMI, education, and other food groups. Excluding those studies not adjusting for energy intake did not change the overall results, suggesting that energy intake may have a limited impact on developing bladder cancer. Second, red and processed meat definition varied across study and this may partially contribute to the observed heterogeneity. Different units of measurements were also used to report risk estimates for meat consumption, and we had to assume average portion sizes when meat consumption was reported as servings. Nevertheless, stratified analysis for different types of measurements and sensitivity analysis for alternative portion sizes did not find substantial differences in results. Third, it was not possible to investigate the association between different meat-cooking methods and bladder cancer because only three articles reported such information. However, none of them found an increase in bladder cancer risk with any of the cooking methods. Fourth, statistical heterogeneity was observed in our analysis as in the previous two reviews [5, 6] but was mainly explained by different study design. After stratification, moderate heterogeneity was still observed among case–control studies, while cohort studies provided more homogenous results.

In conclusion, results from this dose–response meta-analysis suggest that processed meat consumption may be positively associated with risk of bladder cancer. Positive association between red meat and risk of bladder cancer was observed only in case–control studies, while no association was observed in prospective studies.

## Electronic supplementary material

Below is the link to the electronic supplementary material.

Online Resource 1 Dose–response relation between red meat consumption and risk of bladder cancer, assuming a linear-response model in random-effects meta-analysis. The dotted line represents the predicted curve arising from a restricted cubic spline model. The solid line represents the linear trend and the dashed lines its confidence limits. The median value of the lowest reference category (15 g/day) was used as referent. The relative risks are plotted on the log scale.

Online Resource 2 Dose–response relation between processed meat consumption and risk of bladder cancer, assuming a linear-response model in random-effects meta-analysis. The dotted line represents the predicted curve arising from a restricted cubic spline model. The solid line represents the linear trend and the dashed lines its confidence limits. The median value of the lowest reference category (5 g/day) was used as referent. The relative risks are plotted on the log scale.
