# Supplementary figures and images for: Red and processed meat consumption and risk of bladder cancer: a dose–response meta-analysis of epidemiological studies
Source: Eur J Nutr. 2016 Dec 22;57(2):689–701. doi: 10.1007/s00394-016-1356-0 (PMC5845591; doi:10.1007/s00394-016-1356-0)

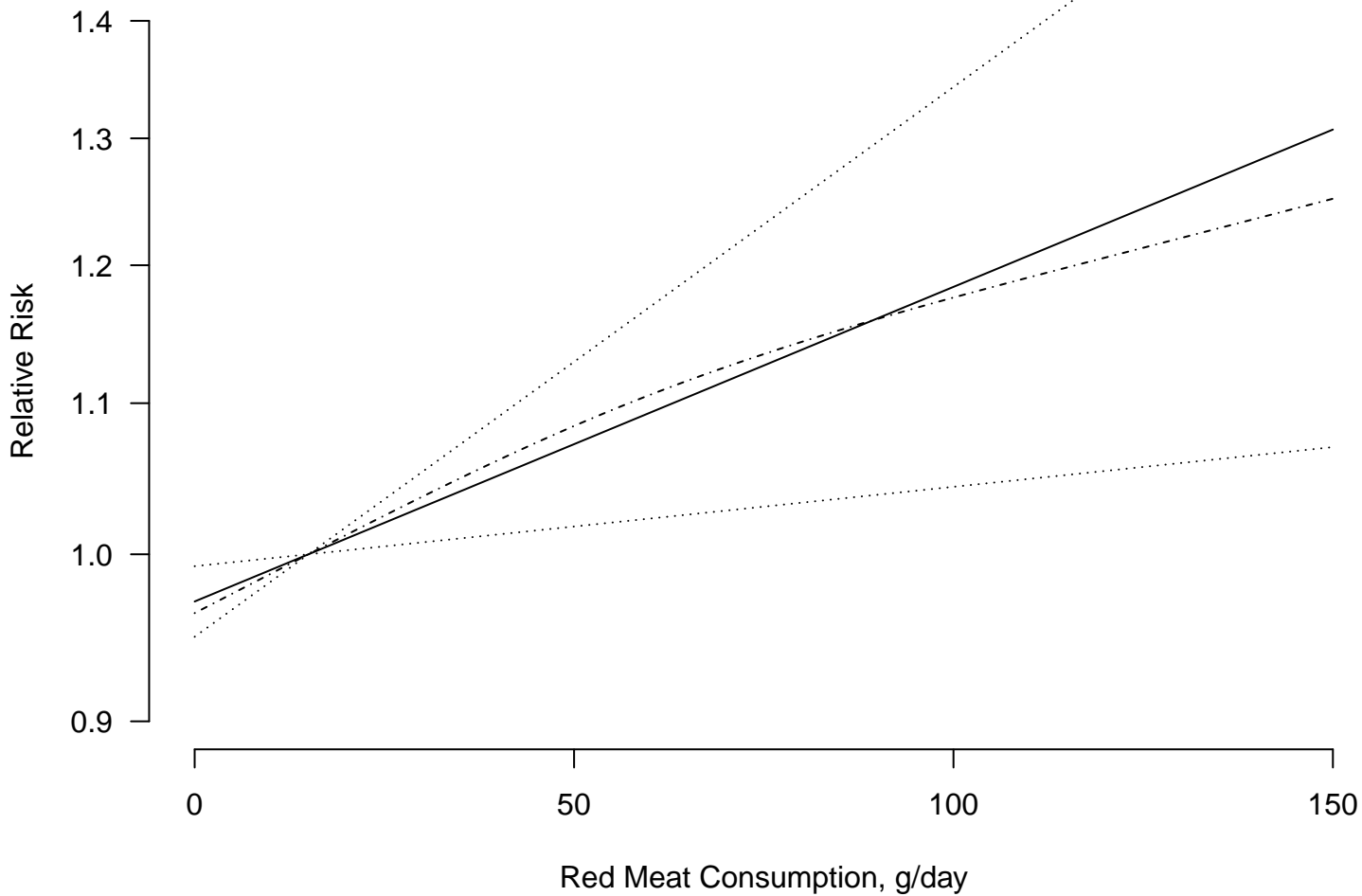

Supplement: Supplementary file 1 — Dose–response relation between red meat consumption and risk of bladder cancer, assuming a linear-response model in random-effects meta-analysis. The dotted line represents the predicted curve arising from a restricted cubic spline model. The solid line represents the linear trend and the dashed lines its confidence limits. The median value of the lowest reference category (15 g/day) was used as referent. The relative risks are plotted on the log scale. [file 394_2016_1356_MOESM1_ESM.pdf]

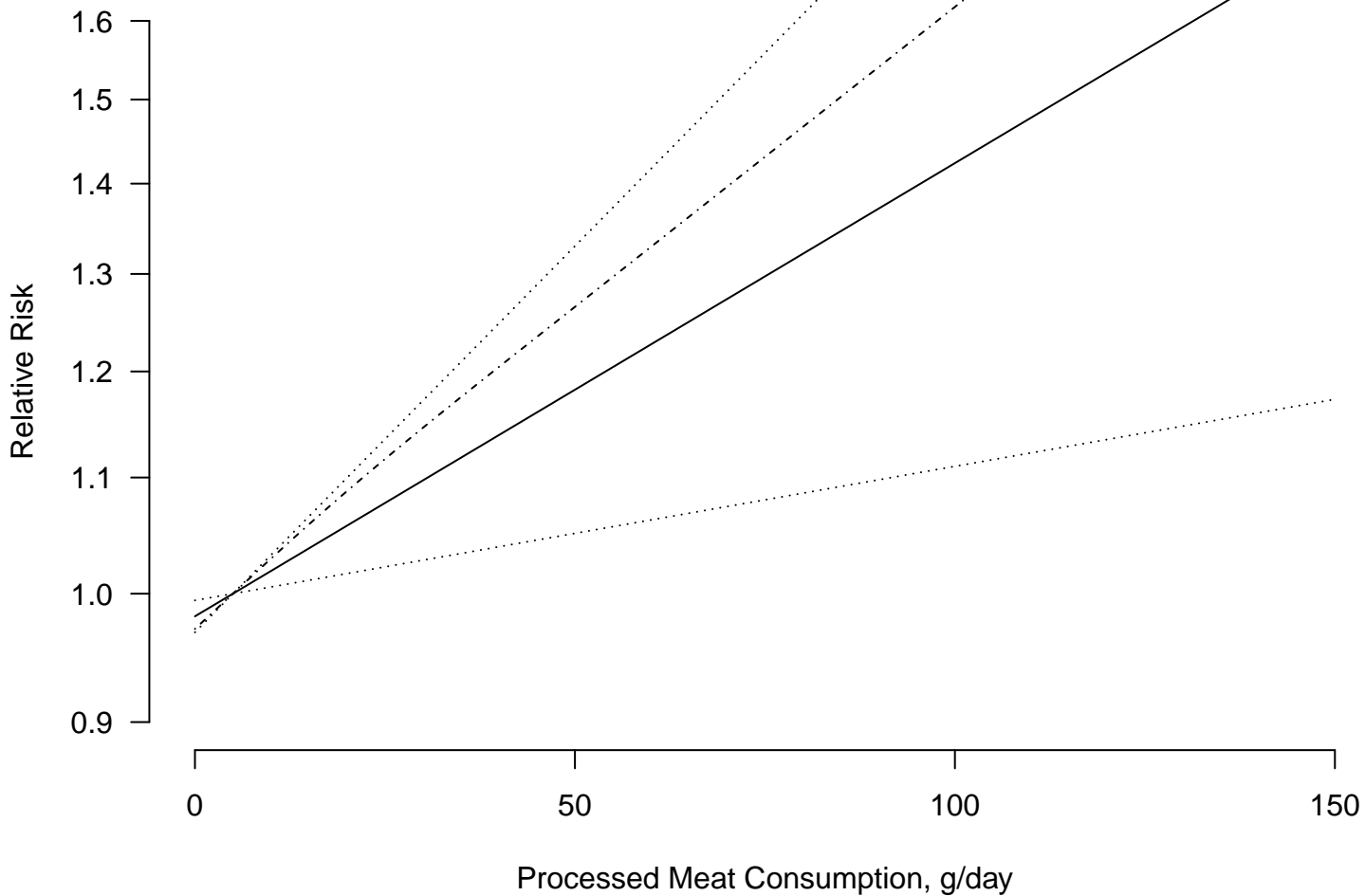

Supplement: Supplementary file 2 — Dose–response relation between processed meat consumption and risk of bladder cancer, assuming a linear-response model in random-effects meta-analysis. The dotted line represents the predicted curve arising from a restricted cubic spline model. The solid line represents the linear trend and the dashed lines its confidence limits. The median value of the lowest reference category (5 g/day) was used as referent. The relative risks are plotted on the log scale. [file 394_2016_1356_MOESM2_ESM.pdf]
